# Validation of the French version of the Functional, Communicative and Critical Health Literacy scale (FCCHL)

**DOI:** 10.1186/s41687-018-0027-8

**Published:** 2018-02-07

**Authors:** Youssoufa M. Ousseine, Alexandra Rouquette, Anne-Déborah Bouhnik, Laurent Rigal, Virginie Ringa, Allan ‘Ben’ Smith, Julien Mancini

**Affiliations:** 1 Aix-Marseille Univ, INSERM, IRD, UMR912, SESSTIM, Institut Paoli-Calmettes, “Cancers, Biomedicine & Society” group, 232, Bd Ste Marguerite, BP 156, 13273 Marseille Cedex 9, France; 2 Public Health and Epidemiology Department, APHP, Bicêtre Hospital, Le Kremlin-Bicêtre, France; 3 Université Paris-Saclay, Univ. Paris-Sud, UVSQ, CESP, INSERM, Le Kremlin Bicêtre, France; 4 Centre for Oncology Education and Research Translation (CONCERT), Ingham Institute for Applied Medical Research & South Western Sydney Clinical School, University of New South Wales, Liverpool, NSW Australia; 5 Psycho-Oncology Co-operative Research Group (PoCoG), School of Psychology, University of Sydney, Sydney, Australia; 6 APHM, Timone Hospital, Public Health Department (BIOSTIC), Marseille, France

**Keywords:** Health literacy, Measurement, Validation studies, Psychometrics, Cancer, France

## Abstract

**Background:**

Health literacy is a key asset, defined as the capacity to acquire, understand and use information in ways which promote and maintain good health.

**Objectives:**

To assess the reliability and validity of the French translation of the Functional, Communicative and Critical Health Literacy (FCCHL) scale.

**Methods/participants:**

A cross-sectional survey using an online questionnaire was proposed to all members of *Seintinelles* association. Exploratory and confirmatory factorial analyses were conducted.

**Results:**

Data from 2342 respondents (45.8% had cancer history) were analysed. The FCCHL scale was well-accepted (missing value by item ≤0.7%). Factor analysis revealed an acceptable fit of three-factor model (comparative fit index = 0.922, root mean square error of approximation = 0.065 and standardized root mean square residual = 0.052). The FCCHL showed satisfactory reliability (α = 0.77) and scalar invariance was reached for education and deprivation, but not for age. Known group validity was verified as mean scale scores differed according to education, deprivation and age, as expected.

**Conclusion:**

The French version of the FCCHL provides a brief reliable and valid measure to explore the dimensions of health literacy. It could be used by health professionals to screen for health literacy level in order to develop this skill and to tailor health communication.

**Electronic supplementary material:**

The online version of this article (10.1186/s41687-018-0027-8) contains supplementary material, which is available to authorized users.

## Introduction

Health literacy (HL) is a key asset, defined as “the individuals' capacity to obtain, process and understand basic health information and services needed to make appropriate health decisions” [1, 2]. Consistently, authors like Nutbeam [3] distinguish three skills: functional literacy, which includes basic skills in reading and writing necessary to understand health information; communicative literacy, which corresponds to the necessary advanced skills to communicate or interact with the healthcare system; and critical literacy to analyse the information obtained to act at best. Accordingly, the Functional, Communicative and Critical Health Literacy scale (FCCHL) measures all three distinguished dimensions of HL [4]. A generic tool [5] was adapted from the first version developed to specifically evaluate HL in diabetic patients [4] and has been validated in several populations including Dutch/German citizens [6–8], and Australian Adolescents and Young Adults with cancer [9]. This brief instrument (14-items) has demonstrated relevance to patients and ease of administration [9]. However, no validation of FCCHL exists in French.

Validated translations of HL measures are needed, as a growing literature has shown the importance of evaluating HL in both patients and general population. Indeed, limited HL predicts poorer health and has demonstrated associations with several patient-reported outcomes, such as poorer health-related quality of life and more mental distress among cancer patients [10].

Our aim was the psychometric evaluation of the French translation of the FCCHL scale.

## Methods

The FCCHL was translated from English to French by three independent researchers. The final version of each item was then chosen with the help of a bilingual psychologist. Individual cognitive interviews were then conducted with six cancer patients to evaluate the wording and understanding of the translated items. Minor refinements were then made comparing our translation with the translation from the Japanese to French made by other French researchers.

Data were collected using a self-administered online questionnaire proposed to all adult members of *Seintinelles* (www.seintinelles.com) between June 16th and 30th, 2016. *Seintinelles* is a French national association including 12,747 members (participant numbers as declared on January 15th 2016) who are cancer patients, cancer survivors and/or other people (e.g. caregivers) “wishing to help cancer research” [11]. They are mostly breast cancer patients because it was initially created based on the model of *Army of Women®*. Use of *Seintinelles* enabled the rapid recruitment of a large sample for psychometric validation of the French translation of the FCCHL scale. The final sample size was larger than the minimum of 500 to 800 respondents needed to perform exploratory factorial analysis for a three-factor scale with 10 to 15 items [12].

Floor or ceiling effects at the scale level were considered to be present if more than 15% of respondents achieved the lowest or highest possible score, respectively [13]. At the item level, these effects were considered to be present if more than 95% answered the lowest or highest response category [14]. Reliability was assessed by Cronbach’s alpha (α) with values α ≥0.7 considered satisfactory [15].

An exploratory factor analysis (EFA) was performed on one third of the sample, randomly selected. To assess construct validity, confirmatory factor analysis (CFA), based on the factorial structure found using EFA and in literature, was subsequently conducted on the other two-thirds of the sample. Multiple-group CFA and nested model comparisons were used to evaluate measurement invariance across age groups, education levels and deprivation using the following sequence: configural, metric and scalar invariance. The robust weighted least squares (WLSMV) estimator was used for both EFA and CFA [16, 17]. The root mean square error of approximation (RMSEA, good fit if <0.06, poor fit if ≥0.10, acceptable elsewhere), the comparative fit index (CFI) and the Tucker-Lewis index (CFI and TLI, good fit if >0.95, poor fit if <0.90, acceptable elsewhere) were used to examine model fit [18]. For measurement invariance, each level of invariance was considered to be met if the fit indices difference between each level was equal or less than −0.01 for ΔCFI and equal or less than 0.015 for ΔRMSEA [19, 20]. When a level of invariance was not met, non-invariant items were identified by reviewing modification indices in order to release equality constraints concerning these items until partial invariance was met.

Our a priori hypotheses were that a higher HL would be associated with higher education, younger age, French as mother language and lack of deprivation [4, 21, 22]. Student’s t tests and ANOVAs were used to compare mean FCCHL levels.

All analyses were two-tailed and performed using SPSS PAWS Statistics 18.0 and Mplus software version 7.4. *P*-values <0.05 were considered significant.

## Results

In June 2016, 2444 participants were surveyed after excluding 124 participants (4.8%) who only completed their sociodemographic characteristics. Missing values by item ranged from 0.1% (FCCHL6) to 0.7% (FCCHL3 and FCCHL5) and 2342 participants (95.8%) answered all 14 items of the FCCHL scale. The following results were obtained from participants with complete data of FCCHL (*n* = 2342).

The mean age of those 2342 participants was 47.6 years (SD = 13.6), 96.4% of participants were women, 45.8% had a history of cancer and 18.1% were deprived (Table 1).

**Table 1:** Main participants’ characteristics (*n* = 2342)

Sociodemographic and medical history		n	%
Age			
18–40		777	33.2
41–60		1111	47.4
61–83		454	19.4
Female Gender		2258	96.4
French maternal language		2269	97 .8
Education level			
Primary/secondary		279	11.9
≤ Three-years higher education		958	40.9
> Three-years higher education		1105	47.2
Deprivation (EPICES Index)		409	18.1
Cancer history		1073	45.8
Difficulties in asking physicians questions			
Always (1)		37	1.6
Often (2)		229	9.8
Sometimes (3)		735	31.4
Rarely (4)		669	28.6
Never (5)		632	27
Missing values		40	1.7
Health literacy	Possible range	Mean	SD
FCCHL	14–70	55.58	7.06
Functional dimension	5–25	18.95	4.18
Communicative dimension	5–25	20.66	3.01
Critical dimension	4–20	15.96	3.08

*FCCHL* Functional, Communicative and Critical Health Literacy

The lowest total score was 27 (versus a possible 14) and 32 respondents (1.4%) had the highest score possible (70) indicating no floor or ceiling effect at the scale level. The percentages of respondents for each of the response categories range from 1% to 72% over the 14 items, indicating no floor or ceiling effects at the item level. Globally, correlations between the different items within each dimension were >0.4 (ranging from 0.41 to 0.76) (Additional file 1: Table S1).

The EFA with promax rotation revealed three factors, explaining 55% of the variance. The loading matrix obtained is shown in Table 2. Among the four items for critical HL, three clearly loaded on the third factor but the last item (FCCHL14) loaded on the second factor (0.42).

**Table 2:** Factor structure of the FCCHL scale. (*n* = 781, Training sample)

		Factor			Communality
		1	2	3	
Functional health literacy					
FCCHL1	Find characters that I cannot read	**0.75**	−0.01	0.08	0.66
FCCHL2	Feel that the print is too small for me to read	**0.59**	−0.09	−0.08	0.43
FCCHL3	Feel that the content is too difficult for me to understand	**0.93**	0.03	0.02	0.88
FCCHL4	Feel that it takes a long time to read them	**0.70**	0.02	−0.01	0.50
FCCHL5	Need someone to help me read them	**0.66**	0.03	−0.05	0.46
Communicative health literacy					
FCCHL6	Collect information from various sources	0.02	**0.78**	−0.04	0.66
FCCHL7	Extract the information I want	−0.03	**0.71**	−0.03	0.52
FCCHL8	Understand the obtained information	0.24	**0.62**	0.06	0.48
FCCHL9	Communicate my opinion about my illness	−0.08	**0.73**	0.08	0.46
FCCHL10	Apply the obtained information to my daily life	0.02	**0.68**	0.04	0.45
Critical health literacy					
FCCHL11	Consider whether the information is applicable to me	−0.02	0.23	**−0.71**	0.73
FCCHL12	Consider whether the information is credible	−0.01	−0.07	**−0.97**	0.86
FCCHL13	Check whether the information is valid and reliable	0.01	0.09	**−0.68**	0.54
FCCHL14	Collect information to make my healthcare decisions	−0.04	**0.42**	−0.27	0.37

Loading values higher than 0.4 are bolded

**Fig. 1:**
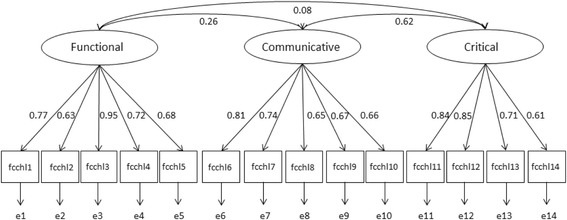
Standardized parameter estimates for the 3-factor model of FCCHL (*n* = 1561, validation sample). Rectangles represent the observed variables (items) and ellipses represent the latent constructs (factors). Values on the single-headed arrows leading from the factors to the items are standardized factor loadings. Values on the curved double-headed arrows are correlations between factors terms

CFA indicated reasonable fit indices for a 3-factor model (with correlation between 3-factors, Fig. 1): RMSEA = 0.087 (90% confidence interval 0.082–0.092), CFI = 0.946 and TLI = 0.933. When the item FCCHL14 was modelled in the second dimension, fit indices were: RMSEA = 0.086 (90% confidence interval 0.081–0.091) and CFI = 0.947 and TLI = 0.935. A significant decrease (3 and 2 factor nested model test, *p* < 0.001) in model fit was observed when communicative and critical dimensions were merged in a single “processing” dimension (RMSEA = 0.123, CFI = 0.888 and TLI = 0.866).

Cronbach’s α were >0.7 for the overall scale (α = 0.77) and subscales (α = 0.79, α = 0.74 and α = 0.77 for functional, communicative and critical dimensions respectively). Communicative and critical HL were moderately linked together (*r* = 0.46) and weakly correlated with the functional dimension (*r* = 0.15 and *r* = 0.03).

Scalar invariance was reached for education levels and deprivation, but not for age (Additional file 1: Table S2). However, partial scalar invariance was reached in releasing the fourth threshold of FCCHL2 in the 18–40 years-old age group. That means that this threshold was significantly lower (0.049) than in the older age groups (0.691), i.e. at the same level of functional HL, older people responded more frequently than younger people that they agreed with the fact that they felt the print was too small for them to read.

Age showed negative associations with FCCHL but not with the communicative dimension (Table 3). When the functional HL or FCCHL score was calculated using the four or thirteen age-invariant items only (without FCCHL2), no association was observed with age (*p*-value = 0.411 or *p*-value = 0.053). A higher education level and lack of deprivation were significantly associated with higher levels of HL, except that deprivation was not associated with critical HL. In contrast having French as mother language (97.8%) was only associated with higher functional HL. No gender differences were observed.

**Table 3:** Bivariate relationships between FCCHL and sociodemographic characteristics (*n* = 2342)

	Functional HL		Communicative HL		Critical HL		FCCHL	
	Mean ± SD	P	Mean ± SD	P	Mean ± SD	P	Mean ± SD	P
Age								
18–40	19.57 ± 3.93	<0.001^a^	20.63 ± 2.78	0.617	16.32 ± 2.75	<0.001	56.53 ± 6.32	<0.001^b^
41–60	18.70 ± 4.27		20.62 ± 3.12		15.86 ± 3.13		55.20 ± 7.34	
61–83	18.49 ± 4.27		20.78 ± 3.15		15.60 ± 3.43		54.88 ± 7.38	
French maternal Language								
No	17.35 ± 4.95	0.006	20.96 ± 2.96	0.472	16.68 ± 2.36	0.094	55.59 ± 7.04	0.548
Yes	18.98 ± 4.16		20.65 ± 3.01		15.95 ± 3.09		55.00 ± 6.95	
Education								
Primary/secondary	17.36 ± 4.36	<0.001	20.11 ± 3.30	<0.001	15.57 ± 3.23	<0.001	53.05 ± 7.47	<0.001
≤ Three-years higher education	18.71 ± 4.12		20.48 ± 2.95		15.63 ± 3.15		54.84 ± 6.78	
> Three-years higher education	19.56 ± 4.05		20.94 ± 2.96		16.34 ± 2.94		56.86 ± 6.92	
Deprivation (EPICES Index)								
No	19.13 ± 4.08	<0.001	20.73 ± 3.02	0.003	15.97 ± 3.06	0.721	55.84 ± 7.00	<0.001
Yes	18.29 ± 4.42		20.25 ± 2.98		15.91 ± 3.17		54.46 ± 7.02	

*HL* Health Literacy, *FCCHL* Functional, Communicative and Critical Health Literacy

^a^*p*-value = 0.411 if functional HL score computed without FCCHL2, item found to be non-invariant across age groups

^b^*p*-value = 0.053 if FCCHL score computed without FCCHL2, item found to be non-invariant across age groups

## Discussion

Our results confirmed similar or better psychometric properties of the French version of the FCCHL scale compared with the original version [5].

Consistent with previous studies [5–7], exploratory analysis revealed a 3-factor model confirming the overall structure of the scale, with satisfactory internal consistency of each FCCHL dimension. Similar to results among German citizens showing a 2-factor model combining communicative and critical HL into “processing HL” [8], these two dimensions were the most correlated here. A single item of the critical dimension (FCCHL14) primarily loaded on the communicative dimension. This dual loading might be explained in light of the wording of this item, because ‘collect’ can reflect accessing information by communication, while ‘make’ refers more to critical aspects needed for making decisions. As no major differences in fit indices were observed when FCCHL14 was included in the communicative dimension, this item was left in the critical dimension to be more consistent with the original version.

The internal consistency of each FCCHL dimension was satisfactory. Our findings differ slightly from previous findings in Dutch citizens [6] and young Australian cancer patients [9], which found that internal consistency of the communicative dimension was less satisfactory (α = 0.63 in both studies). These differences may be explained by fewer items (*n* = 3) in the FCCHL-AYAC for the communicative dimension and difficulties answering items reported by Dutch citizens. In the latter study, items seemed to be too abstract and citizens clearly underlined that they did not understand what was meant by ‘applying information to their daily life’ or by ‘considering whether information was applicable’.

As hypothesized and in line with previous studies [4, 21, 22], people with lower education had lower HL compared to people with higher education. Furthermore, socioeconomic deprivation tended to be associated with lower HL, except for the critical dimension as previously reported [4, 22]. Only the functional dimension of FCCHL was able to discriminate the few participants who had a mother language other than French highlighting difficulties understanding health information associated with potential difficulties understanding French generally.

The negative association between age and functional HL or FCCHL total score disappeared when those scores were computed without the FCCHL2 item found to be non-invariant across age groups. This is likely due to differing interpretations of this item between younger and older people and highlights that spurious age differences may be observed when FCCHL2 is used. It also questions the association between age and FCCHL that was observed in a previous study [4] and suggests that measurement invariance across age should also be studied for FCCHL versions in other languages, as recommended in the guidelines [23]. In our study, the critical HL score was thus the only one that was significantly negatively associated with age and it might reflect lower empowerment among older people [24]. The lack of impact of age on the communicative dimension was expected [4]. It might also be explained by our recruitment of persons, very involved in research and registered to participate in mainly online surveys, who might not have difficulties communicating no matter their age.

Our study has some limitations. We were unable to compare the translated FCCHL against an objective test of functional HL. Moreover, our sample included mainly women (96%), who were highly educated and had French as their mother language. However the sample was more heterogeneous regarding age and cancer history and the FCCHL showed variability despite them being negatively skewed (Additional file 1: Figure S1). FCCHL also presented good acceptability, as indicated by low levels of missing data, which might be attributable to respondents’ high HL.

In this first validation study of a self-report multidimensional health literacy scale in France, the French version of the FCCHL demonstrated adequate reliability and validity among cancer patients and general population. It highlighted that measurement invariance across age should be studied more systematically when validating HL measures. Further studies are needed to examine the French FCCHL stability among less educated, less literate samples including more men and those without French as their mother language. This relatively brief measure could be used among both patients and general population to allow identification of people with low HL in order to develop this skill and to tailor health communication accordingly.

## Additional file

Additional file 1: Table S1. Distribution of the responses to the different items on the questionnaire and polychoric correlations between the various FCCHL items (*n* = 2342). **Table S2.** Measurement invariance across age groups, education levels, and deprivation. **Figure S1.** Distribution of FCCHL score and subscores. (DOCX 77 kb)
