# Supplementary material for: Validation of the French version of the Functional, Communicative and Critical Health Literacy scale (FCCHL)
Source: J Patient Rep Outcomes. 2018 Feb 7;2:3. doi: 10.1186/s41687-018-0027-8 (PMC5934921; doi:10.1186/s41687-018-0027-8)

| **Table S1. Distribution of the responses to the different items on the questionnaire and polychoric correlations between the various FCCHL items (n=2342)** | | | | | | | | | | | | | | | |
| --- | --- | --- | --- | --- | --- | --- | --- | --- | --- | --- | --- | --- | --- | --- | --- |
|  |  | FCCHL1 | FCCHL2 | FCCHL3 | FCCHL4 | FCCHL5 | FCCHL6 | FCCHL7 | FCCHL8 | FCCHL9 | FCCHL10 | FCCHL11 | FCCHL12 | FCCHL13 | FCCHL14 |
| Mean score | | 3.24 | 3.62 | 3.8 | 3.7 | 4.6 | 4.46 | 4.18 | 4.09 | 3.89 | 4.04 | 4.11 | 3.98 | 3.96 | 3.91 |
| Standard deviation | | 1.28 | 1.29 | 1.04 | 1.21 | 0.75 | 0.76 | 0.91 | 0.79 | 1.02 | 0.81 | 0.87 | 1.02 | 1.07 | 1.07 |
| Strongly disagree N (%) | | 451 (19.3) | 788 (33.6) | 669 (28.6) | 757 (32.3) | 1676 (71.6) | 30 (1.3) | 61 (2.6) | 16 (0.7) | 83 (3.5) | 29 (1.2) | 46 (2.0) | 79 (3.4) | 107 (4.6) | 81 (3.5) |
| Disagree N (%) | | 714 (30.5) | 610 (26.0) | 912 (38.9) | 719 (30.7) | 470 (20.1) | 51 (2.2) | 79 (3.4) | 69 (2.9) | 159 (6.8) | 68 (2.9) | 109 (4.7) | 179 (7.6) | 164 (7.0) | 206 (8.9) |
| Not sure N (%) | | 323 (13.8) | 346 (14.8) | 437 (18.7) | 357 (15.2) | 136 (5.8) | 57 (2.4) | 195 (8.3) | 322 (13.7) | 382 (16.4) | 349 (14.9) | 170 (7.3) | 238 (10.2) | 247 (10.5) | 330 (14.1) |
| Agree N (%) | | 662 (28.3) | 452 (19.3) | 277 (11.8) | 415 (17.7) | 41 (1.8) | 887 (37.9) | 1048 (44.7) | 1209 (51.6) | 1020 (43.6) | 1233 (52.6) | 1229 (52.5) | 1063 (45.4) | 1020 (43.6) | 933 (39.8) |
| Strongly agree N (%) | | 192 (8.2) | 146 (6.2) | 47 (2.0) | 94 (4.0) | 19 (0.8) | 1317 (56.2) | 959 (40.9) | 726 (31.0) | 698 (29.8) | 663 (28.3) | 788 (33.6) | 783 (33.4) | 804 (34.3) | 789 (33.7) |
| FCCHL1 | Find characters that I cannot read* | 1 |  |  |  |  |  |  |  |  |  |  |  |  |  |
| FCCHL2 | Feel that the print is too small for me to read* | **0.44** | 1 |  |  |  |  |  |  |  |  |  |  |  |  |
| FCCHL3 | Feel that the content is too difficult for me to understand* | **0.74** | **0.56** | 1 |  |  |  |  |  |  |  |  |  |  |  |
| FCCHL4 | Feel that it takes a long time to read them* | **0.52** | **0.50** | **0.65** | 1 |  |  |  |  |  |  |  |  |  |  |
| FCCHL5 | Need someone to help me read them* | **0.45** | **0.43** | **0.62** | **0.56** | 1 |  |  |  |  |  |  |  |  |  |
| FCCHL6 | Collect information from various sources | 0.05 | 0.08 | 0.16 | 0.11 | 0.18 | 1 |  |  |  |  |  |  |  |  |
| FCCHL7 | Extract the information I want | 0.07 | 0.01 | 0.13 | 0.10 | 0.12 | **0.70** | 1 |  |  |  |  |  |  |  |
| FCCHL8 | Understand the obtained information | 0.28 | 0.11 | 0.36 | 0.28 | 0.25 | **0.44** | **0.44** | 1 |  |  |  |  |  |  |
| FCCHL9 | Communicate my opinion about my illness | 0.12 | 0.06 | 0.15 | 0.12 | 0.12 | **0.43** | **0.41** | **0.48** | 1 |  |  |  |  |  |
| FCCHL10 | Apply the obtained information to my daily life | 0.11 | 0.10 | 0.15 | 0.13 | 0.10 | **0.45** | 0.39 | **0.44** | **0.54** | 1 |  |  |  |  |
| FCCHL11 | Consider whether the information is applicable to me | 0.00 | 0.09 | 0.10 | 0.05 | 0.08 | **0.47** | **0.42** | 0.30 | 0.33 | **0.39** | 1 |  |  |  |
| FCCHL12 | Consider whether the information is credible | 0.00 | 0.05 | 0.04 | -0.01 | 0.02 | 0.40 | 0.35 | 0.22 | 0.23 | 0.30 | **0.76** | 1 |  |  |
| FCCHL13 | Check whether the information is valid and reliable | 0.02 | 0.03 | 0.08 | 0.04 | 0.03 | **0.41** | 0.32 | 0.26 | 0.29 | 0.32 | **0.56** | **0.66** | 1 |  |
| FCCHL14 | Collect information to make my healthcare decisions | 0.08 | 0.02 | 0.10 | 0.07 | 0.06 | 0.40 | 0.35 | 0.32 | **0.43** | 0.36 | **0.44** | **0.42** | **0.42** | 1 |
| Bold characters denotes r>0.40 | | | | | | | | | | | | | | | |
| * Reverse coded in such a way a higher score represented higher HL level | | | | | | | | | | | | | | | |

| **AGE** | | | | | |
| --- | --- | --- | --- | --- | --- |
| **Measurement Invariance model** | **Chi-square (DF)** | **CFI** | **RMSEA (90% CI)** | **ΔCFI** | **ΔRMSEA** |
| **M1: Configural*** | 1095 (222) | .948 | .087 (.082-.092) |  |  |
| **M2: Metric**** | 1133 (244) | .947 | .084 (.079-.089) | -.001 (ΔM1-M2) | -.003 (ΔM1-M2) |
| **M3: Scalar***** | 1408 (322) | .935 | .081 (.076-.085) | -.012 (ΔM2-M3) | -.003 (ΔM2-M3) |
| ** 🠦M3bis: Partial scalar****** | 1350 (321) | .938 | .078 (.074-.083) | -.009 (ΔM2-M3bis) | -.005 (ΔM2-M3bis) |
| * same factor structure across groups |  |  |  |  |  |
| ** loadings constrained to be equal across groups | | |  |  |  |
| ***loadings and thresholds constrained to be equal across groups | | | |  |  |
| **** the fourth threshold of the reversed item FCCHL2 (Feel that the print is too small for me to read) was released in the youngest age group ( estimate in the youngest group was 0.049 whereas it was 0.691 in the two other age groups) | | | | | |
|  |  |  |  |  |  |
| **EDUCATION** | | | | | |
| **Measurement Invariance model** | **Chi-square (DF)** | **CFI** | **RMSEA (90% CI)** | **ΔCFI** | **ΔRMSEA** |
| **M1: Configural*** | 1146 (222) | .941 | .089 (.084-.095) |  |  |
| **M2: Metric**** | 1165 (244) | .942 | .085 (.080-.090) | .001 (ΔM1-M2) | -.004 (ΔM1-M2) |
| **M3: Scalar***** | 1301 (322) | .938 | .076 (.072-.081) | -.004 (ΔM2-M3) | -.009 (ΔM2-M3) |
| * same factor structure across groups |  |  |  |  |  |
| ** loadings constrained to be equal across groups | | |  |  |  |
| ***loadings and thresholds constrained to be equal across groups | | | |  |  |
|  |  |  |  |  |  |
| **DEPRIVATION** | | | | | |
| **Measurement Invariance model** | **Chi-square (DF)** | **CFI** | **RMSEA (90% CI)** | **ΔCFI** | **ΔRMSEA** |
| **M1: Configural*** | 992 (148) | .945 | .087 (.082-.092) |  |  |
| **M2: Metric**** | 999 (159) | .945 | .084 (.079-.089) | .000 (ΔM1-M2) | -.003 (ΔM1-M2) |
| **M3: Scalar***** | 1029 (198) | .946 | .075 (.070-.079) | .001 (ΔM2-M3) | -.009 (ΔM2-M3) |
| * same factor structure across groups |  |  |  |  |  |
| ** loadings constrained to be equal across groups | | |  |  |  |
| ***loadings and thresholds constrained to be equal across groups | | | |  |  |

**Table S2. Measurement invariance across age groups, education levels, and deprivation.**

**Figure S1. Distribution of FCCHL score and subscores.**


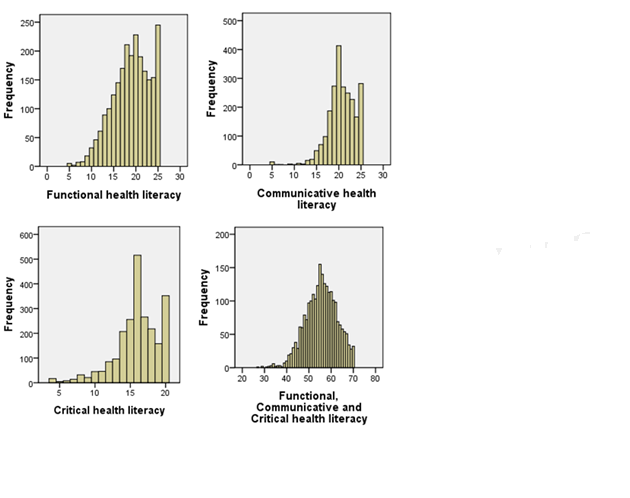

Supplement: Supplementary file 1 — Distribution of the responses to the different items on the questionnaire and polychoric correlations between the various FCCHL items (n = 2342). Table S2. Measurement invariance across age groups, education levels, and deprivation. Figure S1. Distribution of FCCHL score and subscores. (DOCX 77 kb) [file 41687_2018_27_MOESM1_ESM.docx]
